# Coexistence of Anticoagulant and Anti-vascular Calcification Activities in *Kribbella *sp. UTMC 267 Metabolites

**Published:** 2019

**Authors:** Fatemeh Salimi, Javad Hamedi, Elaheh Motevaseli, Fatemeh Mohammadipanah

**Affiliations:** a *Department of Microbial Biotechnology, School of Biology and Center of Excellence in Phylogeny of Living Organisms, College of Science, University of Tehran, Tehran, Iran. *; b *Microbial Technology and Products Research Center, University of Tehran, Tehran, Iran.*; c *Department of Molecular Medicine, School of Advanced Technologies in Medicine, Tehran University of Medical Science, Tehran, Iran.*

**Keywords:** Anticoagulant, Actinobacterial metabolites, Vascular calcification, Warfarin

## Abstract

Thrombotic disorders increase the risk of cardiovascular/cerebrovascular complications and represent a major health problem worldwide. Anticoagulants are extensively used in treatment of these disorders. Vitamin K antagonists, like Warfarin, are frequently used in medication. Vascular calcification (VC) is a significant side-effect of vitamin K antagonists especially Warfarin. There is an urgent need to find natural, efficient, non-toxic, and cost effective anticoagulants without dangerous side-effect like VC. In the present study, we evaluated the potential of thirteen fermentation broth extracts of actinobacteria (FBEA) (200 µg mL^-1^) to prolong whole blood prothrombin time (PT)/international normalized ratio (INR) and activated partial thromboplastin time (APTT). The fractions of the most effective FBEA were further investigated for their inhibitory effect on VC. The results showed PT/INR of the healthy blood samples was sensitive to the presence of five FBEA. Their INR index fell in the 1.2 to 8.6 range and six FBEA prolonged both PT/INR and APTT parameters (1.7-5 INR, and 46-59 s for APTT). The fractions of *Kribbella *sp. UTMC 267 FBE (200 µg mL^-1^), as the most efficient FBE, only inhibited intrinsic and common pathways of coagulation (APTT). Under calcification condition, *Kribbella *sp. UTMC 267 fractions (20 µg mL^-1^) showed significant inhibitory effect on VC in alizarin red staining (13.3-76 %) and alkaline phosphatase activity of VSMCs (33-62%). They also inhibited osteopontin mRNA expression in treated vascular smooth muscle cells (VSMCs) under that situation. So, we can introduce *Kribbella *sp. UTMC 267 FBE as a good candidate for more investigation on thrombotic medication.

## Introduction

According to the most recent world health organization (WHO) report, cardiovascular disease remains the leading cause of death worldwide. Thrombosis is identified as one of the leading disorders for cardiovascular disease like myocardial infarction and other complications including stroke and pulmonary embolism (1). Thus, anticoagulant and antiplatelet agents are effective therapy for prevention or reduction of thrombosis related mortality and morbidity in chronic hemodialysis patients (2). 

There are several anticoagulant drugs with good efficacy; each of them inhibits the coagulation through different mechanisms. These drugs include vitamin K antagonists such as Warfarin, and non-vitamin K antagonist oral anticoagulants (NOACs) like direct thrombin inhibitors and factor Xa inhibitors (3). 

Warfarin is the most known vitamin K antagonist and has been an anticoagulant widely available for human use more than 60 years (4). Although Warfarin has showed an acceptable efficiency in medication, its multiple deleterious side-effects have also been well documented including drug-drug and food-drug interactions, slow dose-adjustment time, hemorrhage and recently recognized vascular calcification (VC) (5, 6). The patients with thrombotic complications who received Warfarin for long period of time suffer from extensive vascular calcification (VC) (7). 

The effect of Warfarin on VC is explained by its inhibitory effect on the central and peripheral carboxylation cycle of several vitamin K-dependent calcification inhibiting factors, such as the matrix gla protein (MGP). Vitamin K is essential for the MGP activation (8). Functional vitamin K deficiency contributes to the high VC burden in haemodialysis patients (9). VC lead to increased prevalence of atherosclerosis, diabetic vasculopathy, chronic kidney disease, myocardial infarction, and hypertension (10-12).

Non-vitamin K antagonist oral anticoagulants (NOACs) are suitable alternatives for vitamin K antagonists, although recent investigation indicated some of their side-effects including increased risk of life-threatening bleeding (13). In addition, NOACs are substrate of cytochrome P450 (CYP) isoenzyme 3A4 and P-glycoprotein (P-gp) transporter. Therefore, co-administration of NOACs with P-gp or CYP3A4 inducers or inhibitors may impact exposure to the NOAC (14-17). Moreover, many rate-controlling and anti-arrhythmic drugs can interference with action of NOACs (16)

All of these reasons prompt for the investigation of natural products with considerable anticoagulant activity and inhibitory effect on VC as attractive alternatives to attenuate VC progression for patients who have consumed Warfarin for a long period of time and now have high burden of VC in comparison to healthy peoples. 

For finding biocompounds with anticoagulant and anti-VC activities, natural sources of potent compounds can be selected for screening program. Among these bio-libraries, actinobacteria are of the best candidates. These bacteria provide useful antibacterial, antifungal (18), antiviral (19), immunosuppressive (20), anticancer (21), anti-diabetes (22), anti-obesity (22) and other pharmacologically effective metabolites. According to our best of knowledge, there is no report on the actinobacterial metabolites with both anti-coagulant and anti-VC activities. Therefore, discovering new anticoagulant compounds with anti-VC activity is becoming increasingly important as more and more because thrombotic complications and VC are directly related to cardiovascular mortality and morbidity worldwide. The goals of the present study were the screening of FBEA for anticoagulant activity, selection of most efficient FBE and its fractionation, purification and finally introduction of an actinobacterium with inhibitory effect on VC. 

## Experimental

*Actinbacterial strains*

Thirteen actinobacterial strains including *Streptomyces* and rare actinomycetes were isolated from different geographical locations in Iran and were deposited in University of Tehran Microorganisms Collection (UTMC) and were used throughout the study.

*Seeding and fermentation processes*

The seed culture (namely FA medium which consists (g L^-1^): starch 10, yeast extract 4, and peptone 2) of each strains was prepared and kept on shaking incubator at 180 rpm and 28 °C for 2 days. Then, 5% (v/v) of the inoculum was transferred to the fermentation medium (FA medium), at 28 °C for 7 days in shaking incubator at 180 rpm. 

*The biocompounds extraction process*

After seven days, the fermentation broth was centrifuged at 2300 *g *for 15 min and the fermentation broth extracts of actinobacteria (FBEA) were obtained from the supernatant using equal volume of ethyl acetate. The solvent phase was evaporated in rotary vacuum evaporator. The obtained FBEA were preserved at -20 °C until use. The extract of un-inoculated FA medium (culture medium extract) was obtained to make sure that putative bioactivities of FBEA are not due to the presence of these compounds in the intact medium. 

*Anticoagulant activities of FBEA*

The effect of FBEA on coagulation parameters including prothrombin time (PT) and activated partial thromboplastin time (APTT) were evaluated. In brief, 100 μL of FBEA (200 μg mL^-1^) were mixed with 100 μL of human plasma and warmed at 37 °C for 180 s. Then, 200 μL of thromboplastin reagent was loaded and PT was measured. For APTT measurement, the mixture of 100 μL FBEA and 100 μL of human plasma were warmed at 37 °C for 60 s, then 100 μL of APTT reagent was added. It was activated for 300 s and APTT was measured after the addition of 100 μL of 25 mM CaCl_2 _(23).

*Fractionation and purification of the most efficient FBE*

Eighty liters of the most efficient FBE, according to its anticoagulant properties, was produced in FA medium. The most efficient FBE was extracted using column chromatography with Amberlite resin (XAD-16) and methanol as stationary and mobile phase, respectively. The obtained FBE was chromatographed over a Silica gel (230-400 mM mesh size) column (90 × 5 cm) and was eluted with mixture of dichloromethane and methanol (10:0, 8:2, 7:3, 6:4, 5:5 and 0:10 v/v). Five hundred milliliters of the fractions were collected at a flow rate of 0.6 mL min^-1^. All the fractions were concentrated using a rotary evaporator. The fractions were mixed based on the similarity of thin layer chromatography profile on TLC silica gel 60F_254_ (Mecherey-Nagel), with dichloromethane and methanol (93:7 v/v) as solvent system, and anisaldehyde staining and these fraction mixtures were screened for anti-VC activities. The most efficient fraction was further fractionated using Sephadex resins (LH-20) as stationary and methanol as mobile phases. The final fractions were collected at a flow rate of 120 µL min^-1^. The fractions were analyzed for anti-coagulant and anti-VC activities. 

**Table 1:** Anticoagulation activity of the fermentation broth extract of actinobacteria (200 µg mL-1) using prothrombin time (PT), and activated partial thromboplastin time (APTT) assays. The results expressed in seconds. PT results also expressed as international normalized ratio (INR). PBS was used as blank. * indicated the signiﬁcant anticoagulant activity

**UTMC code of the strains**	**Strain Name**	**PT** **1 ** **(sec)**			**INR** **2**	**APTT** **1 ** **(sec)**
Blank	-	13			1	37
792	*Promicromonospora iranensis*	13			1	37
103	*Nocardiopsis arvandia*	14.1*			1.2	30
102	*Nocardiopsis sinuspersici*	15.1*			1.3	35
533	*Promicromonospora *sp.	14.1*			1.2	32
1143	*Actinophytocola timorensis*	14.1*			1.2	37
2171	*Nocardiopsis *sp.	38.2*			8.6	37
2189	*Streptomyces *sp	13			1	40*
522	*Kribbella *sp.	14.1*			1.2	39*
693	*Kribbella shirazensis*	17*			1.7	58*
751	*Nocardia *sp	17*			1.7	46*
557	*Nocardia soli*	16*			1.5	59*
2243	*Promicromonospora *sp.	29.1*			5	40*
267	*Kribbella *sp.	28*			4.7	48*

Normal PT and APTT value were recorded as 12-14 s and 24-37 s, respectively (23).

INR is the ratio of the treatment›s prothrombin time and the normal mean prothrombin time (1).

**Table 2:** The effect of various concentrations of the fermentation broth extract fractions of *Kribbella *sp. UTMC 267 on viability of vascular smooth muscle cells (A7r5 cell line). DMSO was used as blank

**FBE’s fractions**	**20 (µg mL** **-1** **)**	**50 (µg mL** **-1)**	**100 (µg mL** **-1** **)**	**150 (µg mL** **-1** **)**	**200 (µg mL** **-1** **)**
Viability rate (%) of treated A7r5 cells					
I	107.03	99.48	107.55	104.16	105.53
II	95.4	90.17	106.83	103.19	93.36
I-A	104.17	90.36	93.36	97.39	84.37
I-B	99.87	93.81	99.67	83.92	91.79
I-C	108.33	93.88	85.42	83.92	89.52
I-D	106.51	118.42	122.20	116.21	125.72

**Figure 1:**
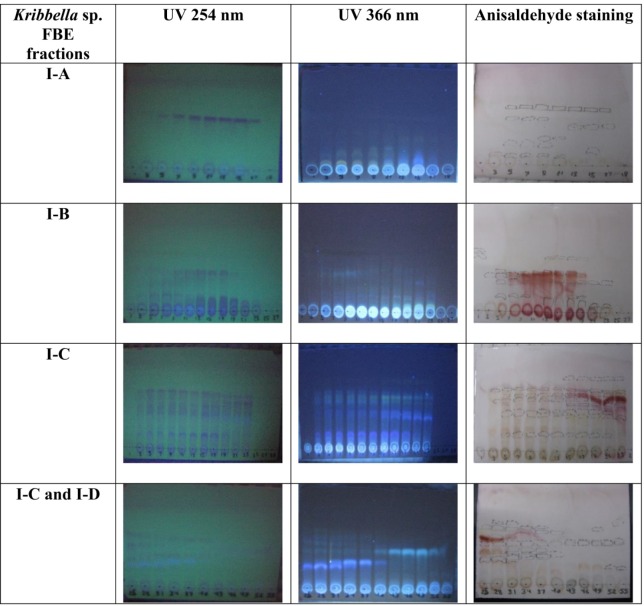
TLC pattern of the fermentation broth extract of *Kribbella *sp. UTMC 267 under UV light (256 and 366 nm) and anisaldehyde staining. The fractions with the same TLC pattern were mixed together, ﬁnally four fractions were obtained and designated I-A, I- B, I-C and I-D

**Figure 2:**
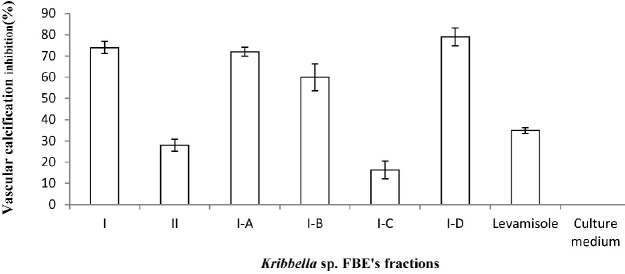
The anti-vascular calciﬁcation effect of the fermentation broth extract fractions of *Kribbella *sp. UTMC 267 (20 µg mL-1). The presence of the calciﬁcation nodules was detected by Alizarin Red staining which attached to the calcium phosphate precipitates. The bars indicated the mean ± standard error (n = 3). The extract of un-inoculated culture medium and Levamisole (20 µg mL-1) were considered as the blank and the positive controls, respectively

**Figure 3:**
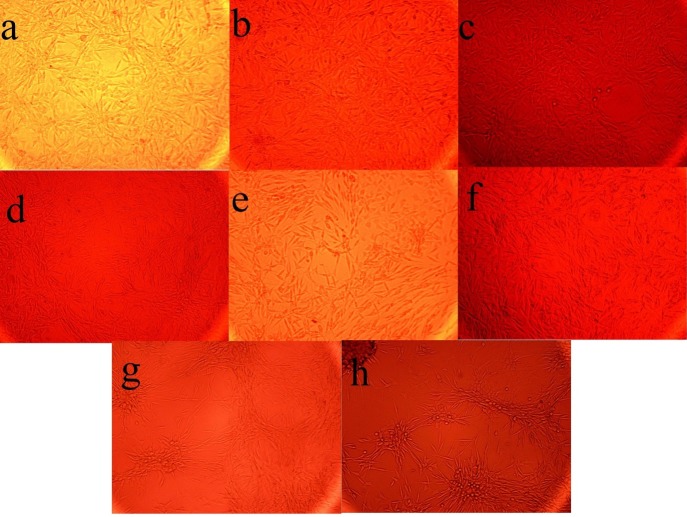
The Effect of the fermentation broth extract (FBE) of *Kribbella *sp. UTMC 267 on the size and the number of calciﬁcation nodules in vascular smooth muscle cells (A7r5). All cells were incubated in the presence of β-glycerophosphate + CaCl2. (a-f) FBE fractions (I-A, I-B, I -C, 1-D, I and II), (g) un-inoculated culture medium extract, (h) no extract was added

**Figure 4:**
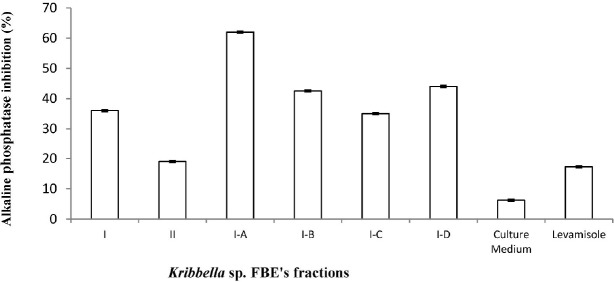
The inhibitory effect of the fermentation broth extract fractions of *Kribbella *sp. UTMC 267 (20 µg mL-1) on alkaline phosphatase (ALP) activity of vascular smooth muscle cell. All cells were incubated in the presence of β-glycerophosphate and CaCl2. Levamisole (20 µg mL**-1**) was used as alkaline phosphatase inhibitor control. The bars indicated the mean ± standard error (n = 3). The inhibitory effect of un-inoculated culture medium extract (20 µg mL**-1**) on ALP activity was also evaluated

*The inhibitory effects of the most efficient FBEA’s fractions on vascular calcification*

To explore the inhibitory effect of the most efficient FBEA’s fractions on vascular calcification, VSMCs (A7r5 cell line, 1×10^4^ cells/ plate) were incubated in 24-well plates containing Dulbecco’s Modified Eagle’s Medium (DMEM) supplemented with 10 mM of sodium pyruvate and 5% FBS in the presence of beta- glycerophosphate (BGP) 

(10 mM) and CaCl_2 _(3 mM) (calcifying medium) to permit maximal mineralization for 14 days. Then, the amount of calcification in A7r5 cells was measured by alizarin red staining and quantitative data were obtained using cetylpyridinium chloride which dissolves attached stain to calcification nodules (24).

Inhibition% = (Absorbance of the calcification group- Absorbance of the treatment group / Absorbance of the calcification group) × 100

*Measurement of ALP activity in presence of the most efficient FBEA’s fractions*

The inhibitory activity of the most efficient FBEA’s fractions on ALP activity of VSMCs was investigated under calcification conditions. The extract of un-inoculated FA medium and Levamisole were used as the blank and the positive control, respectively (25). A7r5 cells (4 × 10^5^ cells/well) were incubated in the calcification condition for 14 days, then the cells were washed with phosphate buffered saline (PBS), and the proteins in the cells were extracted using the lysis buffer (10 mM Tris–HCl, pH 7.5, 100 mM NaCl, 1 mM EDTA, 1 mM EGTA, 1 mL Triton X-100, 5 mL glycerol and 0.01 g SDS in 100 mL ddH_2_O). The alkaline phosphatase (ALP) activity was determined using p-nitro phenyl phosphate as a substrate. The enzymatic activity was normalized to the total protein concentration. The protein was measured using the standard Bradford method (25). 

*The inhibitory effects of the most efficient FBEA’s fractions on osteogenic gene expression*

The effect of the most efficient FBEA’s fractions on the expression of bone-related gene markers in VSMCs under the calcification conditions was determined through real time PCR. Total RNA was isolated from the VSMCs using Trizol (Invitrogen) and reverse-transcribed into cDNA using a cDNA synthesis kit (Takara). The applied primers were as follow: rat osteopontin (OPN) F-5′ CCACAGTCGATGTCCCTGAC3′, R-5′ TGTGGCATCGGGATACTGTT3′, and Runx2 F-5′ GGCCACTTACCACAGAGCTA3′, R-5′AGGCGGTCAGAGAACAAACT3′. 

The relative quantities of the transcripts were determined using a standard curve and were normalized against HPRT (Hypoxanthine-guanine phosphoribosyltransferase). The transcripts were amplified in following cycles: 95 ℃, 15 s; 95 ℃, 15 s; 60 ℃ 15 s, for 40 cycles. Rotor-Gene 6000 Series software version 1.7 and REST software were used for the analysis of the results. 

*Measurement the anti-coagulation activity of the most efficient FBE’s fractions*

Anticoagulation activity of the most efficient FBE’s fractions (200 μg mL^-1^) was evaluated using APTT and PT tests as described, previously. 

*Investigating cytotoxicity of the most efficient FBEA’s fractions using MTT assay*

The cytotoxicity profile of the most efficient FBEA’s fractions on VSMCs (A7r5 cell line) was determined using MTT test (26). The stock solution of the fractions was prepared in DMSO and was diluted by DMEM medium to prepare 20, 50, 100, 150, 200 μg mL^-1^ concentrations of FBEA. The absorbance of the untreated cells (the control group) was considered as 100% viability. The viability of the cells exposed to the most efficient FBEA’s fractions was calculated as follows:

Viability% = (the Absorbance of the Treatment Group / the Absorbance of the Control Group) × 100

## Results

*The Anticoagulant activity of FBEA*

Among tested FBEA, rather than* Promicromonospora iranensis* UTMC 792, twelve FBEA showed anticoagulant activity (Table 1). FBE of *Streptomyces *sp. UTMC 2189 enhanced APTT and had no effect on PT. Both coagulation parameters (APTT and PT) were significantly prolonged in the plasma containing FBE of *Kribbella sp. *UTMC 267 in comparison with that of the normal control plasma. It means that this FBE has the best inhibitory effect on extrinsic, intrinsic and/or ﬁnal common pathways of coagulation, and therefore was selected for further investigations.

*Anti-vascular calcification activity of the Kribbella sp. UTMC 267 FBE’s fractions*

From eighty liters of *Kribbella *sp. UTMC 267 fermentation broth, 20 grams FBE were taken. After loading it in Silica gel containing column and eluting the column with dichloromethane: methanol, several fractions with various polarities were obtained. After observing TLC profile of the fractions, the fractions with the same pattern were mixed together. Finally, two fractions with 0-20% and 30-100% polarities were obtained and designated as I and II fractions, respectively (Figure 1). These fractions (20 µg mL^-1^) showed 72% and 26% anti-VC activity, respectively. Therefore, the fraction I with higher anti-VC activity was further fractioned using Sephadex column chromatography. The obtained fractions (I-A, I-B, I-C and I-D) (20 µg mL^-1^) showed significant anti-VC in the range of 13.3-76 % (Figure 2). The fraction I-D showed the most anti-VC activity. The calcified nodules were developed throughout the cell culture in the calcification group without FBEA (BGP + CaCl_2_). The number and the size of the calcification nodules were considerably reduced in the treatment groups (Figure 3). The extract of un-inoculated FA medium also cannot inhibit vascular calcification and the calcification nodules can be observed (Figure 3).

*The inhibitory effect of the Kribbella sp. UTMC 267 fractions on ALP activity*

ALP activity in the treatment groups was lower than that of in the VSMCs of the calcification group without fractions (p < 0.05). The Levamisole (positive control) and the extract of the intact FA medium (blank) also showed 17.25% and 6.2 5% inhibitory effect on ALP activity, respectively (Figure 4).

*The inhibitory effect of Kribbella sp. UTMC 267 FBE’s fractions on osteoblastic gene expression in VSMCs under the calcification conditions*

In comparison to the calcification group, the expression of OPN was significantly decreased in the fraction I-A and I-B treatment groups by 6.9 and 4 fold, respectively (*P *< 0.05), while, these fractions could not prevent Runx2 mRNA upregulation (*P *> 0.05). The fraction I-C and I-D have no effect on OPN and Runx2 mRNA expression (*P *> 0.05). 

*Anti-coagulation activity of* Kribbella *sp. UTMC 267 fractions*

The results showed that Kribbella sp. UTMC 267 fractions can just have effect on intrinsic and/or ﬁnal common pathways of coagulation cascade because they only enhanced APTT. I-A, I-B, I-C, and I-D fractions delay coagulation in APTT test for seconds (50, 52, 40, and 40 s, respectively) and has no effect on extrinsic pathway which was evaluated by PT test (13 s), suggesting a preferential action toward the intrinsic and/or common pathway of coagulation.

*The evaluation of Kribbella sp. UTMC 267 FBE’s fractions toxicity*

*Kribbella *sp. UTMC 267 FBE fractions at the concentrations of 20-200 µg mL^-1 ^showed limited toxicity. The treated cells showed more than 80% viability in the all applied concentrations. The fraction D showed proliferative activity on A7r5 cell line in all investigated concentrations (Table 2).

## Discussion

Several reports have shown high load of VC in the patients with current or past Warfarin use. This association is consistent with inhibition of MGP. Warfarin blocks MGP carboxylation and its subsequent activation, consequently aggravates VC (9, 27). Therefore, finding natural and cost-effective compounds with both anti-coagulant and anti-VC activities is so critical for the hemodialysis patients who are faced with life-threatening complications related to the coagulation disorders and VC (28). 

The results of this study showed that FBEA have significant potency to inhibit blood coagulation due to their ability to prolong coagulation processes in the samples of human blood which were determined by PT (contact activation pathway) and APTT (tissue factor pathway) in the clinical diagnostic lab-based assays (29, 30). However, their inhibitory mechanisms are not fully understood; because there are some coagulation factors in both pathways. The factors, including II, V, VIII, IX, and XI comprise the intrinsic clotting cascade, and any anticoagulant that affects the activity of these factors can inﬂuence the APTT and the factors, including I, II, III, VII, V and X have also critical role in extrinsic pathway of coagulation, so compounds with inhibitory effect on them can prolong PT (13). 

 It is likely that *Kribbella *sp. UTMC 267 FBE, the most efficient FBE (INR 4.7 and in APTT 48 s), may have inhibitory effect on one or more coagulant factors in extrinsic, intrinsic and/or common pathway of blood coagulation. 

Our experiments proved the presence of biocompounds with anticoagulant activity in the FBEA which can be extracted by ethyl acetate. It means our extraction method is suitable for these biocompounds extraction. These results may be explained by the presence of anticoagulant, anti-thrombotic and fibrinolytic agents in the bacterial extracts especially in the actinobacterial fermentation broth (31, 32). Some recent studies showed that actinobacteria produce compounds with anti-coagulation activities. Coumarin 6-ol, 3,4-dihydro-4,4,5,7-tetramethyl- (CDTM) and 2-(4-Amino-imidazol-[4,5-d]piridazin-1-il)-5-hidroximetil-4-metil-tetrahidrofuran-3-ol are compounds with anticoagulant and anti-thrombotic activities which have been discovered from marine *Streptomyces* sp. and *Salinispora arenicola*, respectively (33, 34).

It has been predicted that no vitamin K antagonist exists in *Kribbella *sp. UTMC 267 FBE fractions because it has been shown that *Kribbella *sp. UTMC 267 FBE’s fractions have inhibitory effect on VC. It seems that they have no inhibitory effect on MGP, which involved in inhibition of vascular calcification. *Kribbella *sp. UTMC 267 fractions (especially I-A, I-B and I-D fractions) obviously decrease the size and number of calcification nodules and other osteogenic marker such as alkaline phosphatase activity of A7r5 cells and OPN mRNA expression in the calcification condition.

In addition*, *it has been shown that *Kribbella *sp. UTMC 267 fractions are not toxic in the concentrations up to 200 µg mL^-1^ on A7r5 cells. The cells in all treatment groups showed more than 80% viability. The incubated cells showed no significant morphological change in all investigated concentrations of *Kribbella *sp. UTMC 267 fractions. The nontoxic property of these fractions can reinforce their therapeutic potential as future anti-thrombosis and anti-VC drug candidates, with particular emphasis on their therapeutic potential in the treatment of cardiovascular and cerebrovascular diseases.

One of the most notable achievements of this study is introducing the metabolites of *Kribbella *sp. UTMC 267 as promising candidates for co-administration with Warfarin to potentiate its anticoagulation activity and decrease its life-threatening side-effect, vascular calcification. Their synergic effect can help in better management of thrombotic disorders with the least side-effects. In conclusion, this study for the first time attempted to indicate the simultaneously presence of pharmacologically active anticoagulant and anti-VC agents in the tested actinobacterial metabolites that could be isolated and evaluated for clinical and physiological applications as natural alternatives. 

*Declaration of conflicting interests*

The authors declare that they have no conflicts of interest. 

*Ethical consideration*

This study has been performed in accordance with ethical standards and in compliance with bioethical rules for working with cell lines and microbial strains. No experiments have yet been conducted on humans or animals for this study. 
